# Combined Therapy with Dacarbazine and Hyperthermia Induces Cytotoxicity in A375 and MNT-1 Melanoma Cells

**DOI:** 10.3390/ijms23073586

**Published:** 2022-03-25

**Authors:** Diana Salvador, Verónica Bastos, Helena Oliveira

**Affiliations:** Department of Biology and CESAM, University of Aveiro, 3810-193 Aveiro, Portugal; diana.s@ua.pt (D.S.); veronicabastos@ua.pt (V.B.)

**Keywords:** melanoma, low-dose chemotherapy, mild hyperthermia, cytotoxicity, cell cycle

## Abstract

Melanoma is a drug-resistant cancer, representing a serious challenge in cancer treatment. Dacarbazine (DTIC) is the standard drug in metastatic melanoma treatment, despite the poor results. Hyperthermia has been proven to potentiate chemotherapy. Hence, this work analyzed the combined action of hyperthermia and DTIC on A375 and MNT-1 cell lines. First, temperatures between 40 °C and 45 °C were tested. The effect of DTIC on cell viability was also investigated after exposures of 24, 48, and 72 h. Then, cells were exposed to 43 °C and to the respective DTIC IC10 or IC20 of each time exposure. Overall, hyperthermia reduced cell viability, however, 45 °C caused an excessive cell death (>90%). Combinational treatment revealed that hyperthermia potentiates DTIC’s effect, but it is dependent on the concentration and temperature used. Also, it has different mechanisms from the treatments alone, delaying A375 cells at the G2/M phase and MNT-1 cells at the S and G2/M phases. Intracellular reactive oxygen species (ROS) levels increased after treatment with hyperthermia, but the combined treatment showed no additional differences. Also, hyperthermia highly increased the number of A375 early apoptotic cells. These results suggest that combining hyperthermia and DTIC should be more explored to improve melanoma treatment.

## 1. Introduction

Melanoma is a deadly tumor that emerges from the transformation of melanocytes. It may occur in both cutaneous and mucosal areas, but over 95% of melanomas are cutaneous [1]. The incidence of this deadly skin cancer has increased over the last three decades [2]. Moreover, the mortality rates of melanoma are rising faster than the majority of the other types of cancer [3].

This type of tumor is highly aggressive and diagnostic at an early stage is a crucial factor for a better prognosis. The outcome depends on the dissemination, thickness, localization, ulceration, and histology of the primary tumor, as well as the patient gender [4]. Early-stage melanoma can be treated with surgery to remove the primary lesion. However, in more advanced stages, such as stage IV melanoma, the prognosis is poor. In fact, melanoma with distant inoperable metastasis is rarely curable [4].

Advanced melanomas usually require chemotherapy. Dacarbazine (DTIC), i.e., 5-(3,3-dimethyltriazeno)imidazole-4-carboxamide, has been the standard chemotherapeutic agent used in melanoma treatment for over 40 years [5]. It was first synthesized in 1959 with the goal of creating a drug capable of interfering with purine biosynthesis [6]. DTIC belongs to the class of the alkylating agents and the subclass of triazenes, being structurally related to purines. It is a pro-drug, requiring conversion in the liver by cytochrome P450 isoforms to the active compound 5-(3-methyl-1-triazeno)imidazole-4-carboxamide (MTIC) [6]. The half-life of MTIC is very short, and this compound decomposes spontaneously into 5-aminoimidazole-4-carboxamide (AIC), an inactive derivative known in purine de novo synthesis, and methyldiazonium cation, the alkylating agent [7]. AIC can be detected in plasma within only 15 min after drug administration, showing that DTIC activation is a very fast process [7]. Exposure to light can also activate DTIC, due to the instability and light sensitivity, resulting in two compounds, 5-diazoimidazole-4-carboxamide and 2-azaipoxantine, that were demonstrated to be cytotoxic in vitro but not in vivo [8,9].

DTIC can be active at all phases of the cell cycle and is not considered a cell cycle phase-specific drug. Its antineoplastic activity is related to the induction of methyl adducts to DNA [7,10]. In fact, O6-methylguanine is the main reason for the cytotoxic effect of DTIC, causing incorrect base pairing [11]. However, the most frequent alkylated site is the N^7^ position of guanine [11]. In addition, there is evidence that methyldiazonium cation can also interact with cellular and soluble proteins and with RNA [12].

This chemotherapeutic agent is administered intravenously in doses of 2–4.5 mg/kg daily for 10 days, repeated at intervals of 4 weeks; or 200–250 mg/m^2^ daily for 5 days, repeated at intervals of 3 weeks; or 850 mg/m^2^ at 3-week intervals [10]. The most common side effects are intense nausea and vomiting, cardiac and hepatic toxicity, myelosuppression, and mucocutaneous toxicity [13,14].

Even though DTIC has the best response rate for melanoma treatment with single chemotherapeutic agents, the response rates are still very low. DTIC has produced response rates between 7% to 25%, with median durations of 5 to 6 months and complete responses of less than 5% [15]. In the search for better responses, some multidrug regimens were tested, being cisplatin, vinblastine and DTIC; and cisplatin, DTIC, carmustine, and tamoxifen the most used. However, polychemotherapy has greater toxicity than single-agent chemotherapy and the data does not show additional clinical benefit compared to DTIC alone [16,17].

Hyperthermia is a therapy discovered centuries ago and it is used to treat diseases by induction of heat. Tumors are treated by being heated to 40–45 °C for a defined period of time [18]. The great advantage of this therapy is that it is generally well tolerated and the effect on normal tissues is minimum or null [19]. Despite its benefits when used alone, hyperthermia is usually applied in combination with other therapies, from which radiotherapy and chemotherapy can be highlighted [20]. In fact, hyperthermia is known to sensitize cancer cells to chemotherapeutic DNA damaging agents [21,22,23]. However, both normal and cancer cells have efficient DNA repair mechanisms that offer protection against therapeutic drugs [24]. Therefore, chemotherapy benefits from hyperthermia given that this therapy considerably affects DNA repair [25]. In addition, hyperthermia can also increase drug uptake by increasing membrane fluidity and permeability and by altering cytoskeleton organization [26,27].

Some in vitro and in vivo studies, as well as clinical trials, have demonstrated that the effect of DTIC can be potentiated by hyperthermia in melanoma treatment [28,29,30]. In this work, we assessed the efficacy of a combined treatment of hyperthermia and low DTIC concentrations to reduce the viability of A375 and MNT-1 cells, both human malignant melanoma cell lines, and investigated the associated toxicity mechanisms. Due to DTIC photosensitivity and instability, rapid decomposition, and short life of DTIC derivates, non-activated DTIC was used in this work.

## 2. Results

### 2.1. Viability of Melanoma Cells after Different Hyperthermic Temperatures

**Figure 1:**
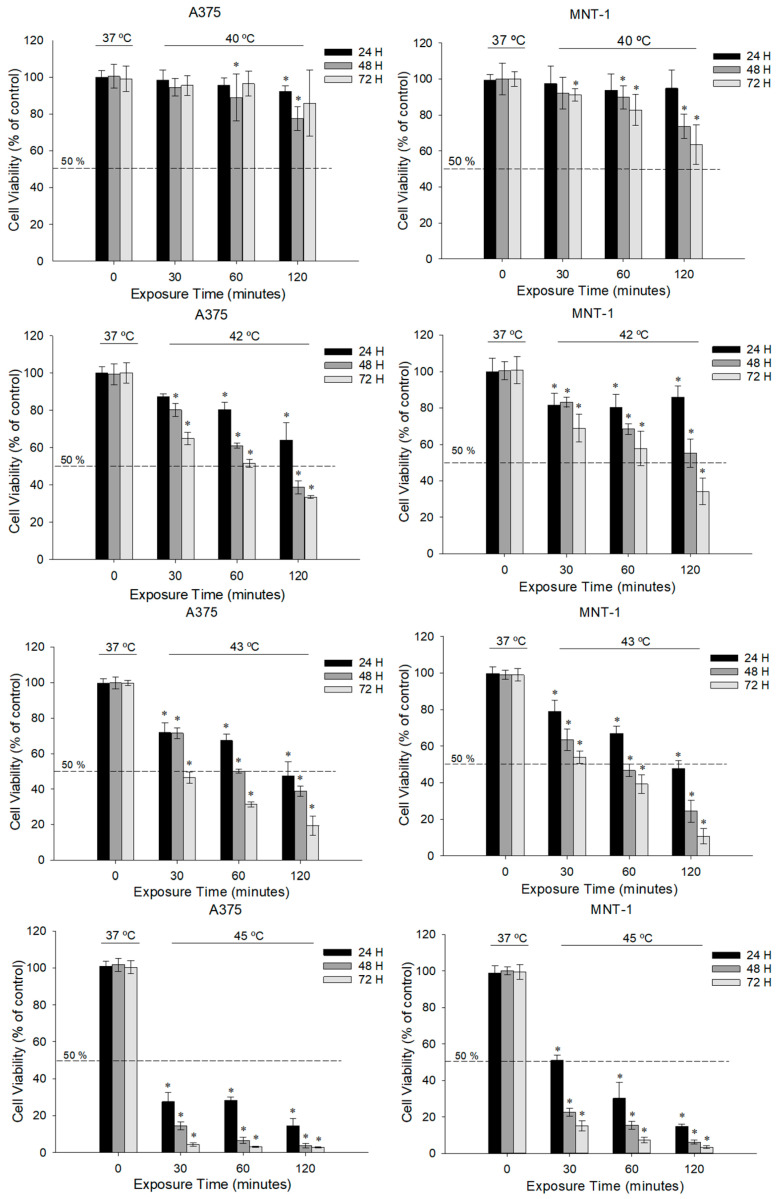
Effect of hyperthermia on the viability of A375 and MNT-1 cells. Cells were exposed to 40 °C, 42 °C, 43 °C or 45 °C for 30, 60, and 120 min and cell viability was determined following 24, 48, and 72 h post-incubation at 37 °C, using MTT assay. Data shown are mean values ± standard deviation of two independent experiments, except 45 °C, which represents only one experiment, with four technical replicates each. * indicates statistical significance in comparison to the respective control condition (*p* < 0.05).

In this series of experiments, we investigated the ideal conditions for hyperthermic exposures in the human malignant melanoma cell lines A375 and MNT-1. In order to assess the most effective temperature for tissue hyperthermia, cells were exposed to different temperatures and cell viability was determined by MTT assay. Data in Figure 1 showed that both cell lines were affected by temperatures above 37 °C. When exposed to 40 °C, there was a significant viability decline in A375 after 120 min exposure and 24 h post-exposure, but no change was observed in the case of MNT-1. Moreover, after 48 h post-exposure, there was a 27% and 32% decline in A375 cells and 11% and 27% decline in MNT-1 cells exposed for 60 and 120 min, respectively. However, no significant change was registered at any condition after 72 h post-exposure for A375 cells, but all exposure times significantly affected MNT-1 cell viability, achieving a maximum reduction of 40%. Furthermore, exposure at higher temperatures induced a more noticeable decrease in both cell lines’ viability. Moreover, all tested conditions significantly and gradually reduced cell viability, except 30 min exposure 24 h post-incubation in the case of A375 cells when exposed to 42 °C. Results showed that even the 30 min exposure to 43 °C was able to reduce A375 viability to 72% and MNT-1 viability to 79%, after 24 h. When exposed to 45 °C, the viability decreased drastically (a maximum reduction of 97% for A375 and 94% for MNT-1 cells), suggesting extreme cellular destruction.

### 2.2. Effect of Exposure to DTIC on Cell Viability

**Figure 2:**
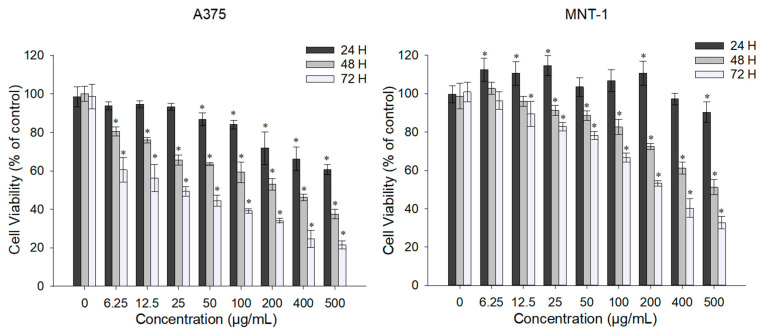
Effect of DTIC on the viability of A375 and MNT-1 cells. Cells were exposed to different concentrations of DTIC for 24, 48, and 72 h and cell viability was determined using MTT assay. Data shown are the mean values ± standard deviation of two independent experiments with four technical replicates each. * indicates statistical significance in comparison to the respective control (*p* < 0.05).

The effect of DTIC in cell viability was evaluated in both cell lines after 24, 48, and 72 h of exposure by MTT assay Figure 2. As expected, DTIC has a concentration-dependent antiproliferative effect. In the case of the A375 cell line, the cells presented cell viability lower than 85% when exposed to DTIC concentrations equal or superior to 100 μg/mL for 24 h. When exposed to this drug for 48 and 72 h, the viability of the A375 cell line was reduced significantly by all tested concentrations. Indeed, the lowest concentration (6.25 μg/mL) was able to reduce cell viability to 80% and to 60% after 48 and 72 h, respectively. In MNT-1 cells, the lowest concentrations tested caused an increase of approximately 10% in cell viability, and only the highest concentration (500 μg/mL) significantly reduced cell viability to 87%, when the exposure time was 24 h. In fact, the lowest concentration showed no significant difference compared to the control even after 72 h exposure. A cell viability reduction of ~80% was obtained with exposure to the concentration of 100 μg/mL for 48 h.

Overall, the DTIC IC_50_ decreased when the exposure time was longer, as shown in Table 1. The A375 cell line was more sensitive to DTIC, showing a more drastic decline to lower concentrations, while MNT-1 showed a more gradual decline. However, A375 had a higher estimated IC_50_ for 24 h exposure compared to MNT-1. Contrary, MNT-1 DTIC IC50 for 72 h exposure was 10-fold higher than A375 IC_50_ for the same exposure time.

Through the obtained values for IC_50_, we calculated the DTIC IC_10_ and IC_20_ of each cell line and each time exposure and applied the acquired concentrations in the following experiments: 38 μg/mL or 138 μg/mL and 477 μg/mL or 538 μg/mL for 24 h, 0.66 μg/mL or 5.5 μg/mL and 45 μg/mL or 115 μg/mL for 48 h, and 0.036 μg/mL or 0.39 μg/mL and 15 μg/mL or 41 μg/mL for 72 h, in the case of A375 or MNT-1, respectively.

### 2.3. Effect of the Combinational Treatment of DTIC and Hyperthermia on Cell Viability

**Figure 3:**
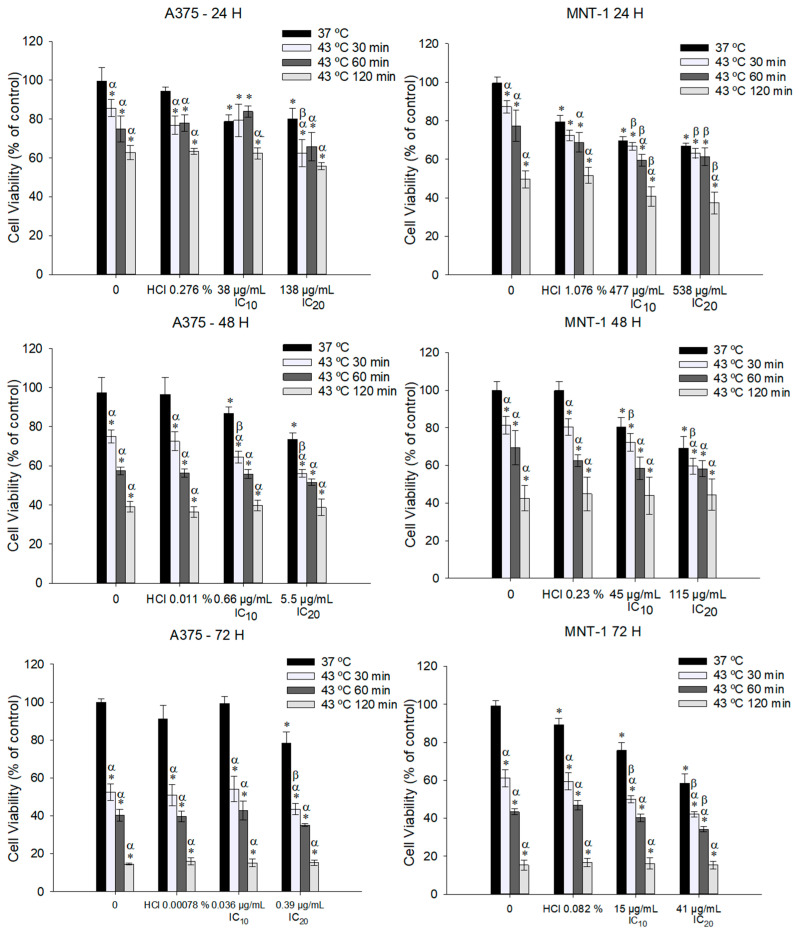
Effect of 43 °C hyperthermia plus DTIC on viability of A375 and MNT-1 cells. Cells were submitted to hyperthermia for 30, 60 or 120 min plus 38 μg/mL or 138 μg/mL and 477 μg/mL or 538 μg/mL during 24 h, 0.66 μg/mL or 5.5 μg/mL and 45 μg/mL or 115 μg/mL during 48 h, and 0.036 μg/mL or 0.39 μg/mL and 15 μg/mL or 41 μg/mL during 72 h, in case of A375 or MNT-1, respectively. Concentrations correspond to the calculated DTIC IC10 and IC20 for each time exposure and for each cell line. HCl concentrations correspond to the equivalent percentage present in DTIC IC20 of each cell line and time exposure. Cell viability was determined using MTT assay. Data shown are the mean values ± standard deviation of two independent experiments, with four technical replicates each. * indicates statistical significance in comparison to the control 37 °C, α indicates statistical significance in comparison to the respective condition at 37 °C and β indicates statistical significance of the conditions with hyperthermia in comparison to hyperthermia alone (*p* < 0.05).

In order to analyze if the effect of DTIC is potentiated by hyperthermia, we exposed A375 and MNT-1 cell lines to 43 °C and to the obtained DTIC IC10 or IC20 of 24, 48, and 72 h exposure. Results revealed that there was at least one combined treatment that significantly reduces cell viability compared to hyperthermia and to DTIC alone in each exposure time tested, as shown in Figure 3. In fact, even when exposed to 30 min at 43 °C and small concentrations as 0.66 μg/mL and 5.5 μg/mL, the A375 cells suffered a more significant reduction in cell viability than when exposed to hyperthermia alone or to DTIC alone, after 48 h. The same was verified for MNT-1 cells, but with the respective DTIC IC20 (115 μg/mL). The smallest concentrations to decrease cell viability were 0.39 μg/mL and 15 μg/mL (A375 and MNT-1, respectively), both combined with 30 min exposure to 43 °C and after 72 h exposure.

### 2.4. Effect of DTIC plus Hyperthermia on Cell Morphology

**Figure 4:**
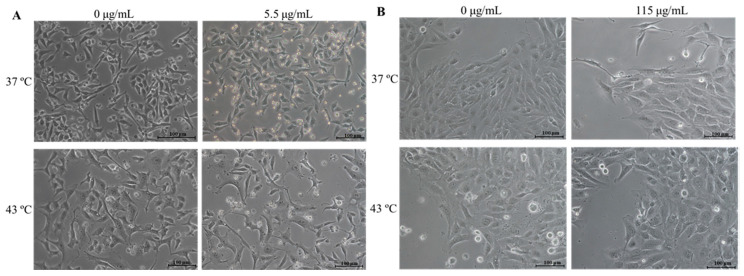
Effect of hyperthermia plus DTIC on morphology of A375 and MNT-1 cells. Cell lines were treated with 43 °C for 30 min and 5.5 μg/mL or 115 μg/mL of DTIC, in case of A375 or MNT-1 cells, respectively. (**A**) A375 cells; (**B**) MNT-1 cells.

The effects of DTIC and hyperthermia alone and in combination on cell morphology are presented in Figure 4. Briefly, cells were exposed to DTIC at the concentration of IC20 of 48 h exposure (A375 to 5.5 μg/mL and MNT-1 to 115 μg/mL), to 43 °C during 30 min or to the treatments combined. As shown in Figure 4A, DTIC alone had more A375 cells in suspension, and hyperthermia alone altered A375 cells morphology, causing the cells to flatten. The combined treatment had a similar effect and some cells also turned round and were less confluent than the control. In the case of MNT-1 cells, changes in morphology were less notable. Nonetheless, some cell roundness was also observed with hyperthermia alone and combined with DTIC. However, the condition with more cells in suspension was hyperthermia alone.

### 2.5. Effect of DTIC plus Hyperthermia on Cell Cycle Distribution

**Figure 5:**
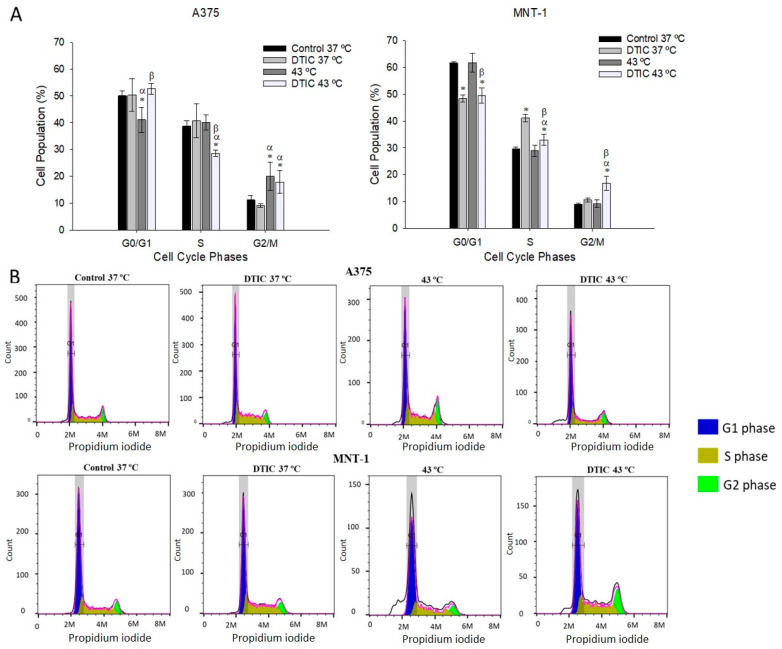
Cell cycle analysis of hyperthermia plus DTIC treated A375 and MNT-1 cells. Cells were exposed to 43 °C for 30 min and 5.5 μg/mL or 115 μg/mL of DTIC, in case of A375 or MNT-1 cells, respectively. (**A**) Cell cycle distribution (%) of both cell lines; (**B**) representative histograms of A375 and MNT-1 cell cycle analysis based on DNA content quantification using propidium iodide staining (arbitrary units). Data shown are the mean values ± standard deviation of two independent experiments, with two technical replicates each, and each replicate with at least 5000 events. * indicates statistical significance in comparison to the control 37 °C, α indicates statistical significance in comparison to the respective condition at 37 °C, and β indicates statistical significance of the conditions with hyperthermia in comparison to hyperthermia alone (*p* < 0.05).

To investigate the possible mechanisms associated with the reduction effect on cell viability, we analyzed the alterations caused by the treatments on cell cycle dynamics. Cells were exposed to 5.5 μg/mL of DTIC in the case of A375 and to 115 μg/mL of DTIC in the case of MNT-1 for 48 h, either alone or in combination with 43 °C hyperthermia for 30 min. A375 cell cycle suffered no change with DTIC alone; however, treatment with hyperthermia alone resulted in a significant decrease in the number of cells at G0/G1 phase from 51% to 41% and increased the percentage of cells at G2/M, as observed in Figure 5. When the treatments were combined, the percentage of A375 cells at the S phase had a significant decrease (9.3%) and the number of cells at G2/M increased, compared to the control. On the contrary, DTIC alone caused a decrease (13%) of MNT-1 cells at the G0/G1 phase and a subsequent increase (11%) in the percentage of cells at the S phase. In MNT-1 cell line, treatment with only hyperthermia had no impact on the cell cycle dynamics. Nevertheless, DTIC plus hyperthermia also decreased MNT-1 cells at G0/G1, resulting in an increase in cells at both S and G2/M phases.

### 2.6. Effect of DTIC plus Hyperthermia on ROS Levels

**Figure 6:**
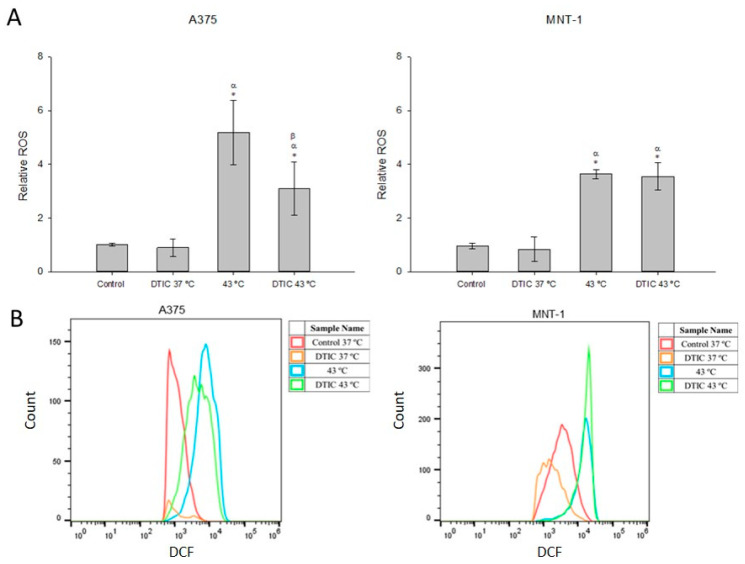
Effects of DTIC combined with hyperthermia on intracellular ROS production. Cells were exposed to 43 °C for 30 min and 5.5 μg/mL or 115 μg/mL of DTIC for 48 h, in case of A375 or MNT-1 cells, respectively. (**A**) Intracellular ROS relative abundance of A375 and MNT-1 cells; (**B**) representative histograms of the abundance of intracellular ROS of both cell lines assessed by the DCF fluorescence intensity (arbitrary units). Data shown are the mean values ± standard deviation of two independent experiments, with three technical replicates each, and each replicate with at least 5000 events. * indicates statistical significance in comparison to the control 37 °C, α indicates statistical significance in comparison to the respective condition at 37 °C, and β indicates statistical significance of the conditions with hyperthermia in comparison to hyperthermia alone (*p* < 0.05).

Intracellular ROS production in A375 and MNT-1 cells exposed to hyperthermia and DTIC was assessed by DCFH-DA. As presented in Figure 6, DTIC alone at concentrations 5.5 μg/mL or 115 μg/mL, in the case of A375 or MNT-1 cells, respectively, did not result in a significant elevation of ROS levels at 48 h. Hyperthermia treatment significantly increased A375 and MNT-1 ROS levels (5.1 and 3.6, respectively), compared to the control. Hyperthermia and DTIC treatments combined also induced a significant increase in the levels of ROS in both cell lines, but in the case of A375, it was 1.7 times lower than hyperthermia alone.

### 2.7. Effects of DTIC plus Hyperthermia on Apoptosis

**Figure 7:**
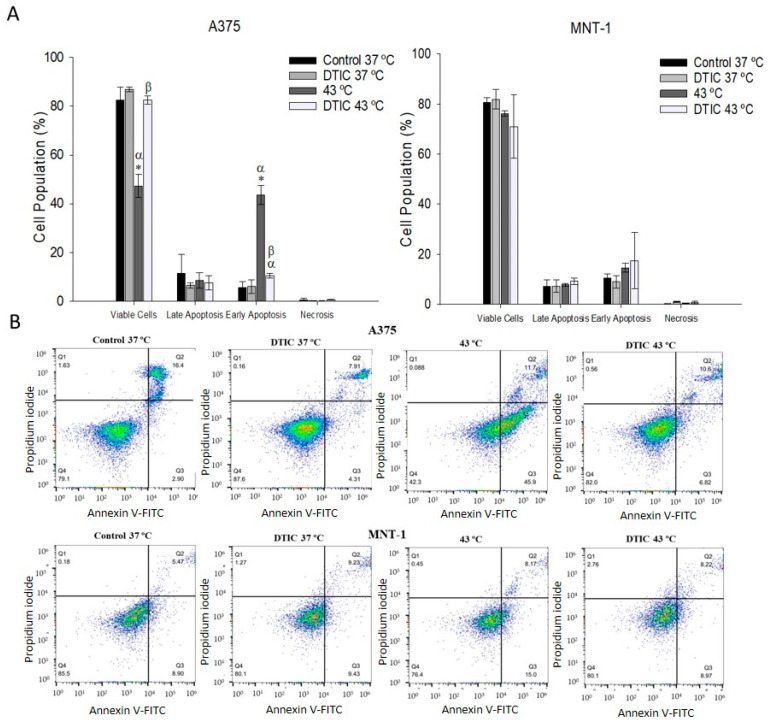
Effect of DTIC and hyperthermia combination on apoptosis in A375 and MNT-1 cell lines. Both cell lines were exposed to 43 °C for 30 min and A375 were treated with 5.5 μg/mL and MNT-1 cells with 115 μg/mL of DTIC for 48 h. (**A**) Apoptotic cells (%) after treatment in groups analogous to viable and non-apoptotic; early and late apoptotic cells; (**B**) representative histograms of Annexin V-FITC/Propidium iodide. Data shown are the mean values ± standard deviation of two independent experiments, with two technical replicates each, and each replicate with at least 5000 events. * indicates statistical significance in comparison to the control 37 °C, α indicates statistical significance in comparison to the respective condition at 37 °C, and β indicates statistical significance of the conditions with hyperthermia in comparison to hyperthermia alone (*p* < 0.05).

The effect of the treatments on cell apoptosis was also measured. The number of A375 viable cells suffered an abrupt decrease when treated with hyperthermia alone, causing an increase in the number of A375 cells in early apoptosis (43%), compared to the control (5.5%). However, neither DTIC alone nor the combined treatment caused an effect in the apoptotic profile of the A375 cell line, as shown in Figure 7. MNT-1 cells’ apoptotic profile was not affected by any of the tested treatments.

## 3. Discussion

Melanoma is an aggressive type of skin cancer responsible for more than 70% of skin cancer-related deaths, being its incidence increasing [31,32]. The heterogeneity of this type of tumor and the low response to treatment of more advanced cases creates a need for novel strategies [33]. Results from diverse clinical studies demonstrated that hyperthermia could enhance the effectiveness of radiotherapy and chemotherapy. Here, we investigated the effects of applying hyperthermia combined with DTIC on A375 and MNT-1 melanoma cells regarding the improvement of melanoma responses to treatment.

The A375 amelanotic cell line carries mutations on BRAF and CDKN2 genes, usually linked to melanoma caused by sun damage [34]. Since the most dangerous environmental factor for melanoma is UV radiation and it is estimated to be responsible for the large majority of cutaneous melanomas, we consider A375 cells a representative and valuable model [35,36]. The MNT-1 cell line is melanotic and has mature stage III and IV melanosomes, which can trap some chemotherapeutic agents preventing nuclear accumulation [37,38]. Moreover, A375 corresponds to a primary melanoma cell line [39], while MNT-1 is a metastatic melanoma cell line [40]. Therefore, analyzing treatments’ effects on these cell lines provides a more suitable representation of the different responses that can encounter in the clinical.

In the present work, we started to analyze the cytotoxic effect of temperatures between 40 to 45 °C in A375 and MNT-1 cell lines in order to determine the temperatures and exposure periods with hyperthermic effect. Upon exposure to 40 °C, the effect on cell viability was barely noticed after 24 h post-exposure, but after 48 h post-exposure A375 viability suffered a maximum decline of 32%. Similarly, MNT-1 cells had a decline of 8–40%, depending on the heating period, after 48 and 72 h post-exposure. Exposure to 42 °C and 43 °C significantly and gradually reduced A375 and MNT-1 viability, causing declines of 14–67% and 14–69% and 28–82% and 18–89%, respectively. These results are consistent with other studies that investigated the effect of hyperthermia in melanoma. In a study with seven human malignant melanoma cell lines (including cell lines that produced melanin) exposed to 42 °C for 4 h, the survival rates of all cell lines were significantly reduced, achieving survival rates between 8.5% and 89%, depending on the cell line [41]. In another work, Shi and colleagues [42], demonstrated that exposure of M21 cells, another human malignant cell line, to 43 °C for 1 h reduced cell viability to about 60%, after 48 h post-exposure. In our investigation, the same conditions led to a decrease in A375 cell viability to 50% and of MNT-1 cell viability to 46%. Mantso and colleagues [28] investigated the effect of temperatures between 37 °C and 50 °C for 2 h in the A375 cell line. The authors concluded that temperatures lower than 43 °C did not affect cell viability, contrary to our results. However, cell viability 24 h post-exposure to 43 °C was reduced 25% and further reduced to 40% when exposed to 45 °C. Moreover, when exposed to 45 °C, cell viability suffered a 90% decline 72 h post-exposure. Similarly, in our work, the temperature at 45 °C caused high cell mortality rates, above 90% after 60 min exposure and 48 h post-exposure in the case of A375 cell line, and after the same exposure period and 72 h post-exposure in the case of MNT-1 cell line.

In order to determine the DTIC concentrations to use in the combinatorial assays, we investigated the effect of DTIC in melanoma cells. The cell viability of both cell lines was significantly decreased in a time- and dose-dependent manner. The effect of DTIC on the cell viability of other human and in mouse melanoma cell lines has already been demonstrated [43,44]. Comparing the cell lines used in the present study, it was noted that MNT-1 cells were less responsive to DTIC than A375 cells, presenting higher ICs. In fact, only the highest concentration tested (500 μg/mL) was able to reduce MNT-1 cell viability after an exposure of 24 h, while A375 cells had the same response with a concentration 10-fold lower. These results may be associated with the high melanin content of MNT-1 cells, considering that melanin has been associated with drug resistance [45].

Further, we intended to analyze the effect of hyperthermia in potentiating the effectiveness of DTIC. According to our primary findings, we determined that 43 °C was the optimal hyperthermic temperature to use in the adjuvant treatment protocol, which is supported by other studies that indicate that combining 40–43 °C with chemotherapy exhibits increased cytotoxicity against cancer cells, including melanoma [46,47]. We exposed both cell lines to 43 °C and to the DTIC IC10 and IC20 of each time exposure (24–72 h) and observed that hyperthermia potentiated the effectiveness of DTIC, although it was dependent on the concentration and heat period applied. Mantso and colleagues [28] also exposed A375 cells to 43 °C for 2 h and to DTIC (5, 10, 30 μM) for 24–72 h. The results are similar to ours, showing that exposing cells to DTIC in combination with hyperthermia had a significantly potentiated effect on reducing cell viability at 48–72 h post-exposure, while at 24 h no significant changes were observed [28]. In our previous work with A375 and MNT-1 cells, we also observed that the potentiated effect of doxorubicin by hyperthermia was dependent on the drug concentration and heat period, demonstrating that not all combined drug and hyperthermia treatments are efficient [21]. Considering our results and with the aim of using a low DTIC concentration and small heating period with a significantly enhanced effect, an exposure time of 30 min to hyperthermia and 48 h to IC20 (5.5 μg/mL to A375 and 115 μg/mL to MNT-1) were selected to the following experiments. These conditions modified cell morphology in a more remarkable way in A375 cells, inducing roundness and flatness, which can be caused by the well-known alterations on cytoskeleton organization by hyperthermia [19].

The cell cycle is usually regulated by checkpoint mechanisms that guarantee genome integrity by causing cell cycle arrest which permits DNA repair or cell death [48,49]. One of the focuses for cancer treatment is cell cycle disruption, through the use of agents that target components of these mechanisms [49]. In our work, the treatments’ effects on cell cycle dynamics were analyzed by flow cytometry. Results showed that treatment of A375 with hyperthermia and hyperthermia plus DTIC induced a significant delay at the G2/M phase. However, while treatment with hyperthermia plus DTIC resulted in a reduction in the percentage of cells at the S phase, hyperthermia alone resulted in a reduction in the percentage of cells in the G0/G1 phase. A similar profile was obtained in another study when B16 cells were exposed to 45 °C for 30 min, resulting in cell arrest at G2/M and a subsequent decrease of cells in G0/G1 [50]. Treatment with DTIC alone had no effect on the A375 cell cycle. In contrast, DTIC alone caused a significant delay of MNT-1 cells at the S phase. A study with B16 and Cloudman S91 cells also revealed that DTIC caused a cell cycle arrest at the S phase but showed a more significant arrest in the G2/M phase [51]. However, this phenomenon was associated with drug concentration increase [51], which can explain the lack of DTIC interference with the A375 cell cycle in our work, since the concentration tested was 5.5 μg/mL. In MNT-1 cells, hyperthermia alone had no impact on the cell cycle. Equally, Orlandi et al. [52] showed that hyperthermia (42 °C for 1 h) had no effect on the cell cycle progression of human melanoma cell lines JR8 and M14. However, in the present work, treatment with hyperthermia plus DTIC caused a significant delay at the S and G2/M phases of MNT-1 cells. These results suggest that the mechanism of the combined treatment is different than the one of the treatments alone and that the effect of hyperthermia alone in cell cycles dynamics is diverse between melanoma cell lines.

Reactive oxygen species production contributes to diverse molecular and biochemical changes involved in the stress response and cancer cell survival [53]. Thus, ROS has been considered an important therapeutic target, being capable of inducing severe cell damage and cell death [54]. The hyperthermia treatment process was also associated with ROS. In fact, a recent study showed that hyperthermia (40 °C for 72 h) is capable of inducing ROS production in the B16-F10 mouse melanoma cell line [55]. Moreover, hyperthermic intraperitoneal chemotherapy, a treatment in which anticancer drugs are heated and then infused and circulate, shows a crucial involvement of ROS [56]. Here, we verified that both cell lines treated with hyperthermia alone or with hyperthermia plus DTIC exhibited an increase in ROS formation. In fact, hyperthermia alone was able to increase more than 5-fold and 3.5-fold ROS production in A375 and MNT-1, respectively. However, in the A375 cell line, the combined treatment led to significantly lower ROS production than hyperthermia alone. In MNT-1 cells, the treatments led to an equal production of ROS. Piotrowska et al. [57], who also analyzed the effect of DTIC in A375 cells, showed an increase in ROS production after 1 h exposure to 6 µM of DTIC, and a decrease after 24 h exposure [57]. Therefore, as we only analyzed the ROS levels at the end of the treatment, corresponding to 48 h exposure, we cannot discard that an increase in ROS production shortly after exposure may have occurred, which may in part explain the caused cellular damage after treatment with DTIC alone and with DTIC plus hyperthermia.

Apoptotic cell death is marked by different physiological changes in cells, such as surface exposure of phosphatidylserine (PS) [58]. PS is a plasma membrane component restrained to the inner membrane leaflet in healthy cells [59]. During the early stages of apoptosis, cell membrane integrity is maintained, but cells lose phospholipid cell membrane asymmetry. PS is translocated to the outer leaflet of the plasma membrane, where it can be measured by fluorescently labeled annexin V conjugates [60]. Annexin V is a preferred probe for PS due to its high calcium-dependent affinity and selectivity for the lipid [61]. Thus, it functions as a marker of the early stage of apoptosis [51]. The effects of 30 min hyperthermia at 43 °C plus DTIC IC20 for 48 h on the induction of apoptosis in A375 and MNT-1 were analyzed. Treatment with hyperthermia alone caused an 8-fold significant increase in A375 early apoptotic cells, compared to the control. Precedent studies have demonstrated that both 43 °C and 45 °C are able to induce apoptosis on B16-F10 and A375 melanoma cell lines through activation of caspase 3 [20,62]. Furthermore, although in MNT-1 cells the combined treatment caused an increase in early apoptotic cells, it was not significant. These results suggest that the induction of apoptosis is not the primary mechanism for the reduced cell viability caused by the combined treatment.

Combinatorial treatments have been showing promising results in melanoma treatment, from which can be highlighted hyperthermia and immunotherapy [63], radiotherapy and immunotherapy [64], and hyperthermia combined with chemotherapy [21]. Here, we demonstrated that combining hyperthermia with DTIC can be a promising alternative to apply in a primary or metastatic melanoma treatment, as shown by A375 and MNT-1 cells, respectively. Nonetheless, the effects of the combined treatment are distinct between cell lines, which can be justified by the fact that these cell lines rely on different metabolic and molecular mechanisms [65]. In fact, A375 cells seem to be more regulated by the c-Jun N-terminal kinase pathway and MNT-1 cells by extracellular signal-regulated kinase activation [65]. Moreover, MNT-1 cells have a more predominant oxidative metabolism than A375 cells [65]. The present study showed that hyperthermia combined with DTIC can reduce cell viability and causes cell cycle delay at the G2/M phase, possibly through the inactivation of mechanisms of DNA repair by hyperthermia [25]. Further studies could be performed to fully understand the involved mechanisms.

## 4. Materials and Methods

### 4.1. Cell Lines and Cell Culture

The amelanotic human melanoma cell line A375 was purchased from the European Collection of Authenticated Cell Cultures (ECACC 88113005) and the pigmented human melanoma cell line MNT-1 was kindly provided by Dr. Manuela Gaspar (iMed.ULisboa, Portugal). Cell lines were maintained in Dulbecco’s Modified Eagle’s Medium (DMEM, Gibco, Life Technologies, Grand Island, NY, USA), supplemented with 10% (*v*/*v*) fetal bovine serum (FBS, Gibco, Life Technologies, Grand Island, NY, USA), 2 mM L-glutamine, 1% pen/strep (100 U/mL penicillin, 100 μg/mL streptomycin, Grisp, Porto, Portugal), and 2.5 μg/mL fungizone (Gibco, Life Technologies, Grand Island, NY, USA). Cells were cultured at 37 °C with 5% CO_2_ and confluence and morphology were frequently monitored. Cells were subcultured at 75–80% confluence.

### 4.2. Determination of Cell Viability

#### 4.2.1. Exposure to Hyperthermia

Cell lines were cultured in 96-well plates at a cell density of 35,000, 20,000, and 10,000 cells/mL for exposures of 24, 48, and 72 h, respectively, and incubated for 24 h at 37 °C with 5% CO_2_ for cell attachment. Next, cells were exposed to different temperatures (40 °C, 42 °C, 43 °C, and 45 °C) for 30, 60, or 120 min in an incubator. The temperature was measured directly in the culture medium in real time. The exposure period started when the cells reached the desired temperature (approximately 30 min for 43 °C). Then, cells were further incubated at 37 °C and cell viability was measured after 24, 48, and 72 h post-exposure. At least two independent assays were performed with 4 replicates each.

#### 4.2.2. Exposure to DTIC

Initially, a stock solution was made dissolving DTIC (S Merck KGaA, Darmstadt, Germany) in hydrochloric acid 1 M (HCl, Merck KGaA, Darmstadt, Germany). Cells were seeded in 96-well plates at the densities mentioned above. The plates were incubated for 24 h at 37 °C with 5% CO_2_ for cell attachment. Then, the cell culture medium was aspirated and replaced by fresh medium with different concentrations of DTIC (6.25, 12.5, 25, 50, 100, 200, 400, 500 μg/mL). Cells were incubated at 37 °C and 5% CO_2_, for 24, 48, and 72 h, and then cell viability was assessed. At least two independent assays were performed with 4 replicates each.

#### 4.2.3. Exposure to Hyperthermia and DTIC

Cells were seeded in 96-well plates as mentioned above and incubated at 37 °C with 5% CO2 for 24 h. Next, medium was aspirated and cells were exposed to DTIC IC10 and IC20 of each exposure time (38 μg/mL or 138 μg/mL and 477 μg/mL or 538 μg/mL for 24 h, 0.66 μg/mL or 5.5 μg/mL and 45 μg/mL or 115 μg/mL for 48 h, and 0.036 μg/mL or 0.39 μg/mL and 15 μg/mL or 41 μg/mL for 72 h, in case of A375 or MNT-1, respectively) and incubated at 43 °C for 30, 60 or 120 min or incubated at 37 °C. Cells submitted to hyperthermia were then transferred to the incubator at 37 °C and cell viability was accessed after 24, 48, and 72 h post-exposure. At least two independent assays were performed with 4 replicates each.

#### 4.2.4. Cell Viability Measurements

Cell viability was determined by the MTT assay (3-(4,5-dimethyl-2-thiazolyl)-2,5-diphenyl-2H-tetrazolium bromide) (9). After treatments, 50 μL of MTT (1.0 mg/mL in phosphate-buffered saline; Merck KGaA, Darmstadt, Germany) was added and the plates were incubated for another 4 h at 37 °C. Then, the medium with MTT was aspirated and 150 μL of dimethyl sulfoxide (DMSO, ≥99.5%, Merck KGaA, Darmstadt, Germany) was added to dissolve the formazan crystals. The plates were shaken in the dark for 2 h and then the absorbance was read in a microplate reader (Synergy HT^®^ Multi-Mode, BioTek^®^, Vinooski, VT, USA) at 570 nm. Cells without exposure to drugs and incubated at 37 °C were used as control. The cell viability was calculated through Equation (1).

(1) $$\mathrm{Cell}\;\mathrm{Viability}\;\left(\%\;\mathrm{of}\;\mathrm{control}\right)=\frac{\mathrm{Sample}\;\mathrm{Absorbance}\;-\;\mathrm{Blank}\;\mathrm{Absorbance}\;}{\mathrm{Control}\;\mathrm{Absorbance}\;-\;\mathrm{Blank}\;\mathrm{Absorbance}}\times 100$$

### 4.3. Cell Morphology

Cells were seeded in 12-well plates at a density of 34,000 cells/mL. After adhesion, A375 and MNT-1 cells were exposed to DTIC at 5.5 or 115 μg/mL, respectively, and to 43 °C for 30 min or incubated at 37 °C. Cells exposed to hyperthermia were then also incubated at 37 °C. After 48 h, cell images were captured using an inverted phase-contrast Eclipse TS100 microscope (Nikon, Tokyo, Japan).

### 4.4. Cell Cycle Analysis

A375 and MNT-1 cells were seeded in 12-well plates at a density of 34,000 cells/mL. After 24 h at 37 °C, the cell culture medium was replaced with fresh medium with DTIC at concentrations equivalent to estimated DITC IC20 at 48 h of exposure of each cell line. Thereafter, the cells were incubated at 37 °C or at 43 °C for 30 min and cultured at 37 °C for 48 h. The cell monolayer was washed with 500 μL of PBS, trypsinized with 150 μL Trypsin-EDTA, and incubated for 5 min at 37 °C. Afterwards, 300 μL of the medium was added and the cells were collected and centrifuged at 700× *g* for 5 min. The supernatant was removed, the cell pellets were washed in PBS, then fixed with 1 mL of 85% cold ethanol and stored at −20 °C until analysis.

At the time of analysis, cells were centrifuged at 112 g for 6 min at 4 °C and ethanol was removed. The pellets were resuspended with 800 μL of PBS and filtered with nylon filter membranes. At this point, 50 μg/mL of RNase (Merck KGaA, Darmstadt, Germany) were added and incubated for 10 min. Next, 50 μg/mL of propidium iodide (PI, ≥94%, Merck KGaA, Darmstadt, Germany) was added and samples were incubated for at least 20 min at room temperature in the dark. Cell cycle distributions were analyzed on an Attune^®^ Acoustic Focusing Cytometer (Applied Biosystems, Thermo Fischer Scientific, Waltham, MA, USA) flow cytometer. Two independent assays with two replicates each were performed for each treatment, and for each sample at least 5000 events were acquired. The percentage of cells in G0/G1, S, and G2/M phases was determined using the FlowJo software (FlowJo LLC, Ashland, OR, USA).

### 4.5. Analysis of Intracellular ROS Levels

The intracellular levels of ROS were measured using the probe 2′,7′-dichlorofluorescein diacetate (DCFH-DA, Merck KGaA, Darmstadt, Germany), which in the presence of ROS is converted into the highly fluorescent 2′,7′-dichlorofluorescein (DCF). Briefly, cells were seeded in 12-well plates at a density of 34,000 cells/mL and incubated with complete culture medium for 24 h. Next, the medium was replaced with fresh medium with the respective concentrations of DTIC (5.5 or 115 μg/mL, in the case of A375 or MNT-1, respectively). Cells were exposed to 37 °C or 43 °C for 30 min, followed by incubation at 37 °C for 48 h. Then, the medium was removed, cell monolayers were washed with 500 μL of PBS, and treated with 10 μM DCFH-DA in culture medium supplemented with 2% FBS. After 30 min incubation at 37 °C, cells were washed, trypsinized, and resuspended in cold DMEM medium containing 2% FBS. DCF fluorescence was analyzed within 45 min by flow cytometry using an Attune^®^ Acoustic Focusing Cytometer (Applied Biosystems, Thermo Fischer Scientific, Waltham, MA, USA). Data analysis was performed using FlowJo software (Tree Star Inc., Ashland, OR, USA).

### 4.6. Cell Apoptosis Assay

Quantitative determination of the apoptosis was performed by flow cytometry using the Annexin V-FITC Apoptosis Detection Kit (BD Biosciences, Franklin Lakes, NJ, USA). A375 and MNT-1 cells were seeded in 6-well plates at a density of 34,000 cells/mL and of 44,000 cells/mL, respectively, and incubated for 24 h. After treatment of 30 min at 43 °C and 5.5 or 115 μg/mL of DTIC for 48 h, for A375 or MNT-1, respectively, cells were gently collected, counted, and washed twice in PBS after centrifugation (300× *g*, 5 min, 4 °C). Then, cells were resuspended in 1x binding buffer and 5 μL of both Annexin V-FITC and PI was added to 100 μL of cell suspension (1 × 10^5^ cells). Cells were then incubated in the dark for 15 min at room temperature and 400 µL of binding buffer was added to each sample. Data were acquired in the following hour on an Attune^®^ Acoustic Focusing Cytometer (Applied Biosystems, Thermo Fischer Scientific, Waltham, MA, USA) and analysis was performed with FlowJo software (FlowJo LLC, Ashland, OR, USA).

### 4.7. Statistical Analysis

All data are represented as the mean ± standard deviation. Data were analyzed statistically using SigmaPlot version 14.0 (Systat Software, San Jose, CA, USA) for Windows. Data were analyzed by one-way ANOVA (*p* < 0.05) followed by Dunnett’s test (*p* < 0.05) in the case of the initial experiment with exposure to only hyperthermia or only DTIC and followed by Tukey’s test (*p* < 0.05) for multiple comparations in the following experiments.

## Figures and Tables

**Table 1:** Inhibitory concentrations (IC_50_) obtained for 24, 48 and 72 h DTIC exposure. Values are expressed in μg/mL. Data are presented as the mean ± standard error.

Cell Line		24 h	48 h	72 h
A375	IC_50_	1158 ± 164.4	205 ± 45.7	22.5 ± 4.35
MNT-1	IC_50_	673.2 ± 157.0	603.4 ± 61.08	227 ± 13.9

## Data Availability

All data reported in this paper are contained within the manuscript.

## References

[1] Yde S.S., Sjoegren P., Heje M., Stolle L.B. (2018). Mucosal Melanoma: A Literature Review. Curr. Oncol. Rep..

[2] Chen Y.-N. (2012). Dacarbazine inhibits proliferation of melanoma FEMX-1 cells by up-regulating expression of miRNA-200. Eur. Rev. Med. Pharmacol. Sci..

[3] Zhu F., Liang Y., Chen D., Li Y. (2016). Melanoma Antigen Gene Family in the Cancer Immunotherapy. Cancer Transl. Med..

[4] Eggermont A.M.M., Kirkwood J.M. (2004). Re-evaluating the role of dacarbazine in metastatic melanoma: What have we learned in 30 years?. Eur. J. Cancer.

[5] Wilson M., Schuchter L., Kaufman H., Mehnert J. (2016). Chemotherapy for Melanoma. Melanoma.

[6] Murray A., Wexler P. (2014). Dacarbazine. Encyclopedia of Toxicology.

[7] Marchesi F., Turriziani M., Tortorelli G., Avvisati G., Torino F., De Vecchis L. (2007). Triazene compounds: Mechanism of action and related DNA repair systems. Pharmacol. Res..

[8] Beal D.D., Skibba J.L., Whitnable K.K., Bryan G.T. (1976). Effects of 5-(3,3-Dimethyl-1-triazeno)imidazole-4-carboxamide and Its Metabolites on Novikoff Hepatoma Cells. Cancer Res..

[9] Shetty B.V., Schowen R.L., Slavik M., Riley C.M. (1992). Degradation of dacarbazine in aqueous solution. J. Pharm. Biomed. Anal..

[10] Al-Badr A.A., Alodhaib M.M., Brittain H.G. (2016). Dacarbazine. Profiles of Drug Substances, Excipients and Related Methodology.

[11] Čimbora-Zovko T., Brozovic A., Piantanida I., Fritz G., Virag A., Alič B., Majce V., Kočevar M., Polanc S., Osmak M. (2011). Synthesis and biological evaluation of 4-nitro-substituted 1,3-diaryltriazenes as a novel class of potent antitumor agents. Eur. J. Med. Chem..

[12] Bull V.L., Tisdale M.J. (1987). Antitumour imidazotetrazines-XVI. Macromolecular alkylation by 3-substituted imidazotetrazinones. Biochem. Pharmacol..

[13] Bhatia S., Tykodi S., Thompson J. (2009). Treatment of Metastatic Melanoma: An Overview. Oncology.

[14] Jiang G., Li R.-H., Sun C., Liu Y.-Q., Zheng J.-N. (2014). Dacarbazine Combined Targeted Therapy versus Dacarbazine Alone in Patients with Malignant Melanoma: A Meta-Analysis. PLoS ONE.

[15] Bedikian A.Y., Millward M., Pehamberger H., Conry R., Gore M., Trefzer U., Pavlick A.C., DeConti R., Hersh E.M., Hersey P. (2006). Bcl-2 antisense (oblimersen sodium) plus dacarbazine in patients with advanced melanoma: The oblimersen melanoma study group. J. Clin. Oncol..

[16] Jilaveanu L.B., Aziz S.A., Kluger H.M. (2009). Chemotherapy and biologic therapies for melanoma: Do they work?. Clin. Dermatol..

[17] Wu S., Singh R.K. (2011). Resistance to chemotherapy and molecularly targeted therapies: Rationale for combination therapy in malignant melanoma. Curr. Mol. Med..

[18] Habash R.W.Y., Romanovsky A.A. (2018). Therapeutic hyperthermia. Handbook of Clinical Neurology.

[19] Chicheł A., Skowronek J., Kubaszewska M., Kanikowski M. (2007). Hyperthermia—Description of a method and a review of clinical applications. Rep. Pract. Oncol. Radiother..

[20] Mantso T., Vasileiadis S., Lampri E., Botaitis S., Perente S., Simopoulos K., Chlichlia K., Pappa A., Panayiotidis M.I. (2019). Hyperthermia Suppresses Post—In Vitro Proliferation and Tumor Growth in Murine Malignant Melanoma and Colon Carcinoma. Anticancer. Res..

[21] Salvador D., Bastos V., Oliveira H. (2022). Hyperthermia Enhances Doxorubicin Therapeutic Efficacy against A375 and MNT-1 Melanoma Cells. Int. J. Mol. Sci..

[22] Hosoya N., Miyagawa K. (2014). Targeting DNA damage response in cancer therapy. Cancer Sci..

[23] Zaffaroni N., Fiorentini G., De Giorgi U. (2001). Hyperthermia and hypoxia: New developments in anticancer chemotherapy. Eur. J. Surg. Oncol..

[24] Goldstein M., Kastan M.B. (2015). The DNA damage response: Implications for tumor responses to radiation and chemotherapy. Annu. Rev. Med..

[25] Oei A.L., Vriend L.E.M., Crezee J., Franken N.A.P., Krawczyk P.M. (2015). Effects of hyperthermia on DNA repair pathways: One treatment to inhibit them all. Radiat. Oncol..

[26] Hurwitz M., Stauffer P. (2014). Hyperthermia, radiation and chemotherapy: The role of heat in multidisciplinary cancer care. Semin. Oncol..

[27] Moyer H.R., Delman K.A. (2009). The role of hyperthermia in optimizing tumor response to regional therapy. Int. J. Hyperth..

[28] Mantso T., Vasileiadis S., Anestopoulos I., Voulgaridou G.P., Lampri E., Botaitis S., Kontomanolis E.N., Simopoulos C., Goussetis G., Franco R. (2018). Hyperthermia induces therapeutic effectiveness and potentiates adjuvant therapy with non-targeted and targeted drugs in an in vitro model of human malignant melanoma. Sci. Rep..

[29] Stojkovic R., Radacic M. (2002). Cell kiling of melanoma B16 in vivo by hyperthermia and cytotoxins. Int. J. Hyperth..

[30] Storm F.K., Kaiser L.R., Goodnight J.E., Harrison W.H., Elliott R.S., Gomes A.S., Morton D.L. (1982). Thermochemotherapy for melanoma metastases in liver. Cancer.

[31] Guy G.P., Thomas C.C., Thompson T., Watson M., Massetti G.M., Richardson L.C., Centers for Disease Control and Prevention (CDC) (2015). Vital signs: Melanoma incidence and mortality trends and projections—United States, 1982–2030. MMWR Morb. Mortal. Wkly. Rep..

[32] Quintanilla-Dieck M.J., Bichakjian C.K. (2019). Management of Early-Stage Melanoma. Facial Plast. Surg. Clin. N. Am..

[33] Shannan B., Perego M., Somasundaram R., Herlyn M., Kaufman H., Mehnert J. (2016). Heterogeneity in melanoma. Melanoma.

[34] Avram S., Coricovac D.E., Pavel I.Z., Pinzaru I., Ghiulai R., Baderca F., Soica C., Muntean D., Branisteanu D.E., Spandidos D.A. (2017). Standardization of A375 Human Melanoma Models on Chicken Embryo Chorioallantoic Membrane and Balb/c Nude Mice. Oncol. Rep..

[35] Brozyna A.A., Hoffman R.M., Slominski A.T. (2020). Relevance of Vitamin D in Melanoma Development, Progression and Therapy. Anticancer Res..

[36] Sample A., He Y.Y. (2018). Mechanisms and Prevention of UV-Induced Melanoma. Photodermatol. Photoimmunol. Photomed..

[37] Chen K.G., Leapman R.D., Zhang G., Lai B., Valencia J.C., Cardarelli C.O., Vieira W.D., Hearing V.J., Gottesman M.M. (2009). Influence of melanosome dynamics on melanoma drug sensitivity. J. Natl. Cancer Inst..

[38] Chen K.G., Valencia J.C., Lai B., Zhang G., Paterson J.K., Rouzaud F., Berens W., Wincovitch S.M., Garfield S.H., Leapman R.D. (2006). Melanosomal sequestration of cytotoxic drugs contributes to the intractability of malignant melanomas. Proc. Natl. Acad. Sci. USA.

[39] Giard D.J., Aaronson S.A., Todaro G.J., Arnstein P., Kersey J.H., Dosik H., Parks W.P. (1973). In Vitro Cultivation of Human Tumors: Establishment of Cell Lines Derived From a Series of Solid Tumors. J. Natl. Cancer Inst..

[40] Cuomo M., Nicotra M.R., Apollonj C., Fraioli R., Giacomini P., Natali P.G. (1991). Production and Characterization of the Murine Monoclonal Antibody 2G10 to a Human T4-Tyrosinase Epitope. J. Investig. Dermatol..

[41] Goss P., Parsons P.G. (1977). The effect of hyperthermia and melphalan on survival of human fibroblast strains and melanoma cell lines. Cancer Res..

[42] Shi Z., Lan B., Peng B., Wang X., Zhang G., Li X., Guo F. (2018). Combination therapy with BH3 mimetic and hyperthermia tends to be more effective on anti-melanoma treatment. Biochem. Biophys. Res. Commun..

[43] Jin J., Gong J., Yin T., Lu Y., Xia J., Xie Y., Di Y., He L., Guo J., Sun J. (2011). PTD4-apoptin protein and dacarbazine show a synergistic antitumor effect on B16-F1 melanoma in vitro and in vivo. Eur. J. Pharmacol..

[44] Woiniak A., Drewa G., Wozniak B., Schachtschabel D.O., Mila-Kierzenkowska C., Drewa T., Olszewska-Slonina D., Soponska M. (2005). The effect of antitumor drugs on oxidative stress in B16 and S91 melanoma cells in vitro. Med. Sci. Monit..

[45] Gidanian S., Mentelle M., Meyskens F.L., Farmer P.J. (2008). Melanosomal Damage in Normal Human Melanocytes Induced by UVB and Metal Uptake—A Basis for the Pro-oxidant State of Melanoma. Photochem. Photobiol..

[46] Hildebrandt B., Wust P., Ahlers O., Dieing A., Sreenivasa G., Kerner T., Felix R., Riess H. (2002). The cellular and molecular basis of hyperthermia. Crit. Rev. Oncol. Hematol..

[47] Issels R.D. (2008). Hyperthermia adds to chemotherapy. Eur. J. Cancer.

[48] Diaz-Moralli S., Tarrado-Castellarnau M., Miranda A., Cascante M. (2013). Targeting cell cycle regulation in cancer therapy. Pharmacol. Ther..

[49] Pavey S., Spoerri L., Haass N.K., Gabrielli B. (2013). DNA repair and cell cycle checkpoint defects as drivers and therapeutic targets in melanoma. Pigment. Cell Melanoma Res..

[50] Wang H.-X., Yang Y., Guo H., Hou D.-D., Zheng S., Hong Y.-X., Cai Y.-F., Huo W., Qi R.-Q., Zhang L. (2016). HSPB1 deficiency sensitizes melanoma cells to hyperthermia induced cell death. Oncotarget.

[51] Olszewska-Słonina D., Styczyńisk J., Drewa T., Olszewski K., Czajkowski R. (2005). B16 and cloudman S91 mouse melanoma cells susceptibility to apoptosis after dacarbazine treatment. Acta Pol. Pharm..

[52] Orlandi L., Zaffaroni N., Bearzatto A., Silvestrini R. (1996). Effect of melphalan and hyperthermia on p34cdc2 kinase activity in human melanoma cells. Br. J. Cancer.

[53] Pelicano H., Carney D., Huang P. (2004). ROS stress in cancer cells and therapeutic implications. Drug Resist. Updates.

[54] Trachootham D., Alexandre J., Huang P. (2009). Targeting cancer cells by ROS-mediated mechanisms: A radical therapeutic approach?. Nat. Rev. Drug Discov..

[55] Mundhara N., Majumder A., Panda D. (2021). Hyperthermia induced disruption of mechanical balance leads to G1 arrest and senescence in cells. Biochem. J..

[56] Wang C., Chen F., Kim E., Harrison L. (2007). Thermal sensitization through ROS modulation: A strategy to improve the efficacy of hyperthermic intraperitoneal chemotherapy. Surgery.

[57] Piotrowska A., Wierzbicka J., Rybarczyk A., Tuckey R.C., Slominski A.T., Żmijewski M.A. (2019). Vitamin D and its low calcemic analogs modulate the anticancer properties of cisplatin and dacarbazine in the human melanoma A375 cell line. Int. J. Oncol..

[58] van Engeland M., Nieland L., Ramaekers F., Schutte B., Reutelingsperger C. (1998). Annexin V-affinity assay: A review on an apoptosis detection system based on phosphatidylserine exposure. Cytology.

[59] Birge R.B., Boeltz S., Kumar S., Carlson J., Wanderley J., Calianese D., Barcinski M., Brekken R.A., Huang X., Hutchins J.T. (2016). Phosphatidylserine is a global immunosuppressive signal in efferocytosis, infectious disease, and cancer. Cell Death Differ..

[60] Kupcho K., Shultz J., Hurst R., Hartnett J., Zhou W., Machleidt T., Grailer J., Worzella T., Riss T., Lazar D. (2019). A real-time, bioluminescent annexin V assay for the assessment of apoptosis. Apoptosis.

[61] Concha N., Head J., Kaetzel M., Dedman J., Seaton B. (1992). Annexin V forms calcium-dependent trimeric units on phospholipid vesicles. FEBS Lett..

[62] Shellman Y.G., Howe W.R., Miller L.A., Goldstein N., Pacheco T.R., Mahajan R.L., LaRue S.M., Norris D.A. (2008). Hyperthermia Induces Endoplasmic Reticulum-Mediated Apoptosis in Melanoma and Non-Melanoma Skin Cancer Cells. J. Investig. Dermatol..

[63] Hurwitz M.D. (2019). Hyperthermia and immunotherapy: Clinical opportunities. Int. J. Hyperth..

[64] Tagliaferri L., Lancellotta V., Fionda B., Mangoni M., Casà C., Di Stefani A., Pagliara M.M., D’Aviero A., Schinzari G., Chiesa S. (2021). Immunotherapy and radiotherapy in melanoma: A multidisciplinary comprehensive review. Hum. Vaccines Immunother..

[65] Brito C., Tomás A., Silva S., Bronze M.R., Serra A.T., Pojo M. (2021). The Impact of Olive Oil Compounds on the Metabolic Reprogramming of Cutaneous Melanoma Cell Models. Molecules.

